# Epicardial cardiac cavernous Haemangioma-a case report

**DOI:** 10.1186/s12872-019-1156-6

**Published:** 2019-07-29

**Authors:** Mahmoud Yousef Ibrahim Abuharb, Xiao Ming Bian, Jian He

**Affiliations:** The first affiliated hospital of Dalian Medical University, Lianhe Avenue, Dalian, China

**Keywords:** Heart neoplasms, Haemangioma, Mitral stenosis, Rheumatic heart

## Abstract

**Background:**

Cardiac haemangiomas are exceptionally rare. They are usually solitary growths. Cardiac haemangiomas can be classified as capillary, cavernous, or arteriovenous in nature. They can occur in any chambers of the heart, but are predominantly found at the intramural or endocardial layers.

**Case presentation:**

This is a rare case of a cardiac haemangioma located on the epicardium of a 52-year-old male patient. The patient complained of 1-year duration of chest tightness and shortness of breath. The haemangioma was removed successfully. For symptomatic lesions, surgical removal remains the preferred treatment.

**Conclusion:**

The pathological diagnosis was primary cardiac cavernous haemangioma. In this case, the haemangioma was successfully resected with invasive surgery. No recurrence was detected on follow up.

## Background

Haemangiomas refer to the type of tumours of blood vessel cell types. They develop from vascular endothelial hyperplasia and are usually benign in nature. Cardiac haemangioma is a very rare type of benign heart tumour, accounting for only 2.8% of all primary cardiac tumours [1]. The autopsy detection rate ranged from as low as 0.0017 to 0.27% in a recent study [2]. The sites of origin of cardiac haemangioma can be from any of the three cardiac layers, either endocardium, myocardium, or epicardium. In some cases, cardiac haemangiomas can also be found across the whole pericardium [3]. Among all, the epicardium is the rarest site of origin for this tumour. In addition, in terms of the anatomic sites of origin, slightly more than one third (36%) of the cardiac haemangiomas are found in the right ventricle, another one-third (34%) were from the left ventricle. Approximately 23% of the cardiac haemangiomas are located in the right atrium (23%) and only very few (7%) cases are found in the left atrium. In terms of vascular classification, there are 3 types of cardiac haemangiomas; namely cavernous tumour with large, thin-walled vascular spaces, arteriovascular tumour which is an unorganized malformation of arteries and veins, and lastly tumour with hypervascular capillaries [4].

The growth mechanism of cardiac haemangioma is not fully understood. It can present simultaneously with other heart diseases, resulting in diagnostic dilemma in certain cases. In cases of primary pericardial neoplasms, patients often present with various types of localized symptoms, such as exertional dyspnoea, chest pain, cough, palpitations; and systemic symptoms including fatigue, night sweats, fever, and facial or lower limb oedema. Among all, the most commonly reported symptom by the patients is exercise intolerance [5]. Nevertheless, it has also been recently reported in the literature on how a primary cardiac haemangioma was detected in a completely asymptomatic patient who presented for an unrelated illness [6]**.**

In view of the wide variety in the symptom presentation, imaging modality is an important component in the investigation of primary cardiac haemangioma. Initial imaging approach such as chest radiography (CXR) or transthoracic echocardiography have limited value. On the other hand, magnetic resonance imaging (MRI) and computed tomography (CT) are more useful as these cross-sectional imaging modalities allow further characterization to the condition, and may, in some cases, provide diagnostic findings. With respect to treatment option, cardiac haemangiomas are not always amenable to surgery. Successful resection can be performed only if the tumour is well circumscribed and small. In this case report, we outline the case of an adult male who presented with epicardial cardiac haemangioma.

## Case presentation

**Fig. 1:**
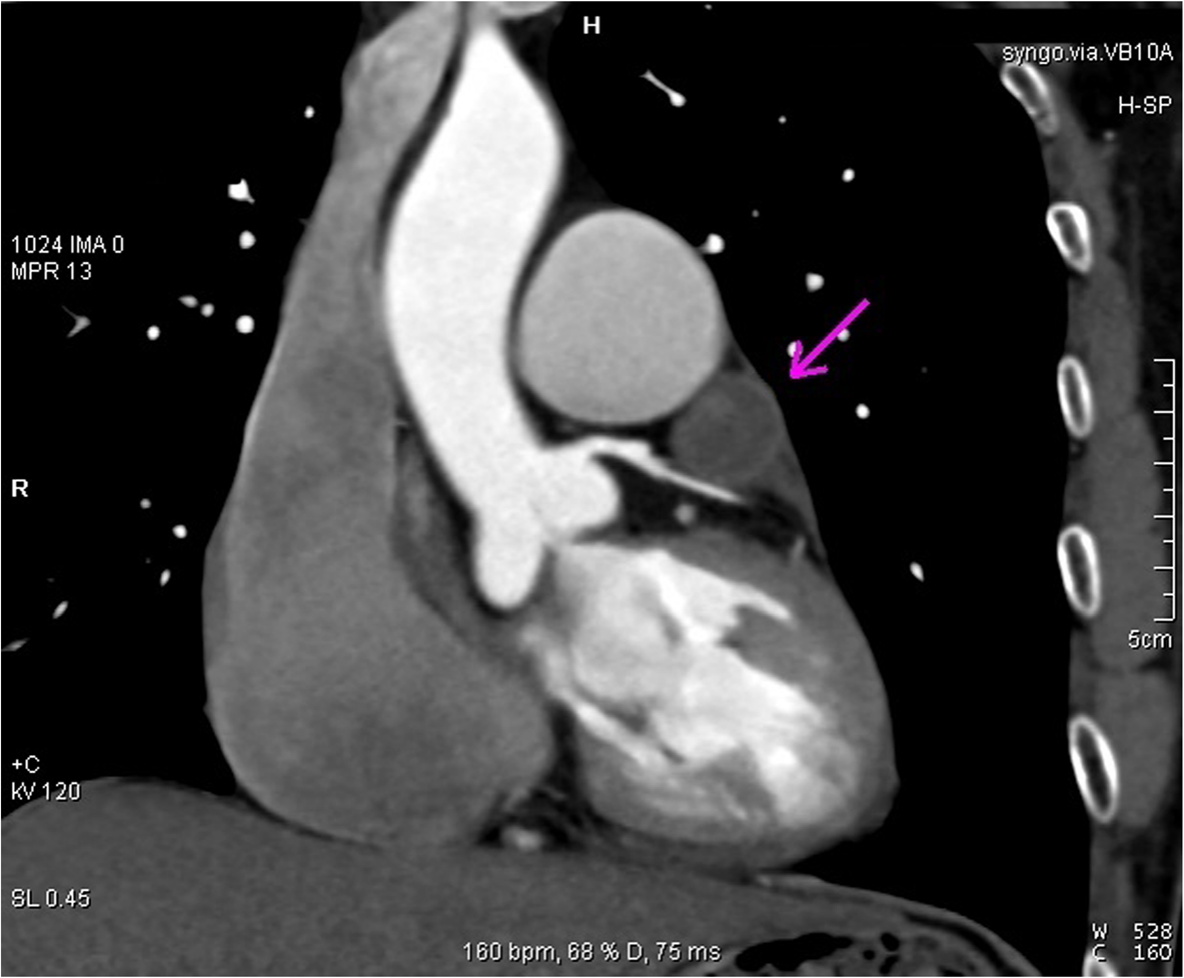
Cardiac 2D CTA reconstruction. The purple arrow points to the mass

**Fig. 2:**
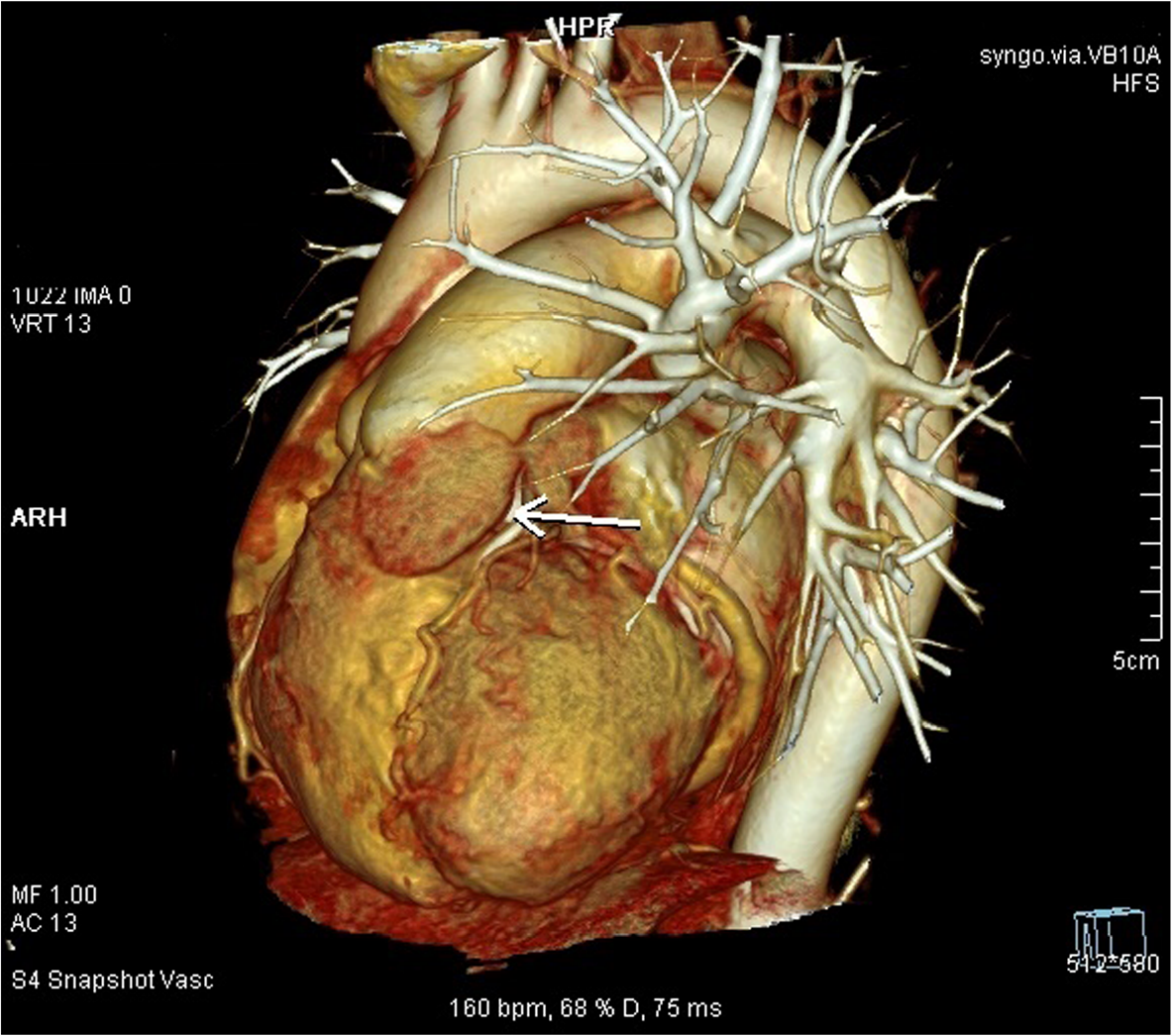
Cardiac 3D CTA reconstruction. The white arrow points to the mass

**Fig. 3:**
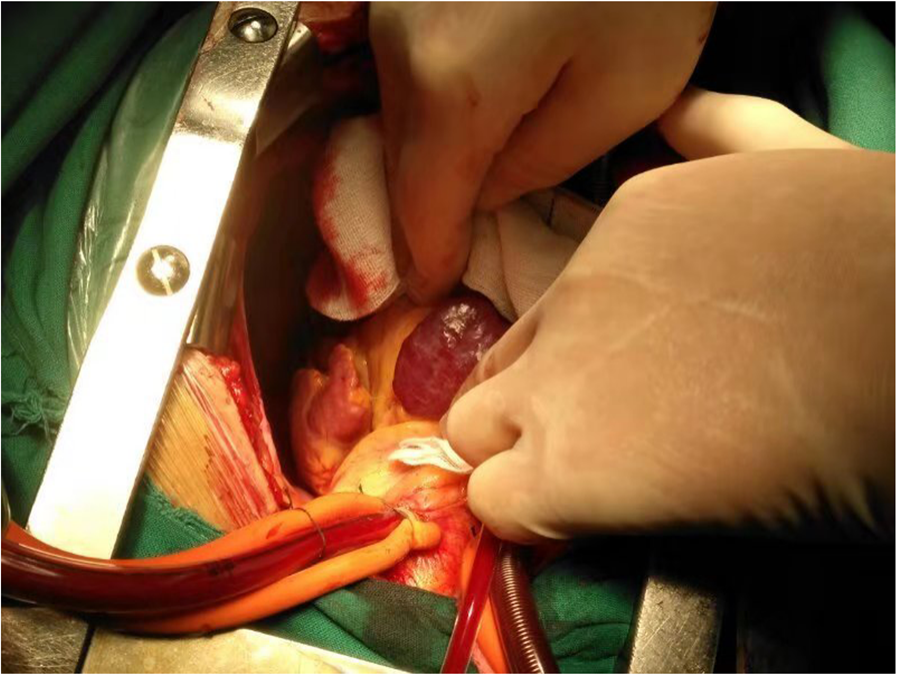
Intraoperative view of the tumour. A heart-shaped mass, was visible on the surface of the anterior wall of the right ventricle. It was approximately 3 × 2 cm in size

A 52-year-old-male patient presented to the hospital on 4th of January 2018 with a 1-year duration of chest tightness and shortness of breath. The symptoms had been worsening for the past 1 month. Physical examination showed BP120/70 mmHg, HR 72/min. Electrocardiography (ECG) showed a heart rate of 72/min, sinus rhythm and an incomplete right bundle branch block. Echocardiography (ECHO) of the heart revealed that the left atrium was 56 × 72 mm, the right atrium was 42 × 56 mm, the left ventricle was 42 mm, the right ventricle was 19 mm. Left ventricular ejection fraction (LVEF) was 56%. Mitral valve disease with valve leaflet thickening, left atrial enlargement, thrombosis over the left atrial anterior wall and left atrial appendage was also noted. Arterial blood gas analysis was normal. Computed tomography angiography (CTA) with iodine based contrast medium was performed. It revealed a 3 × 2 cm mass on the epicardium, located just over the anterior wall of the right ventricular outflow tract (Figs. 1, 2 and 3). All these findings were consistent with the diagnosis of cardiac haemangioma. Patient was also revealed to have underlying rheumatic heart disease, specifically mitral stenosis with regurgitation during the investigation.

**Fig. 4:**
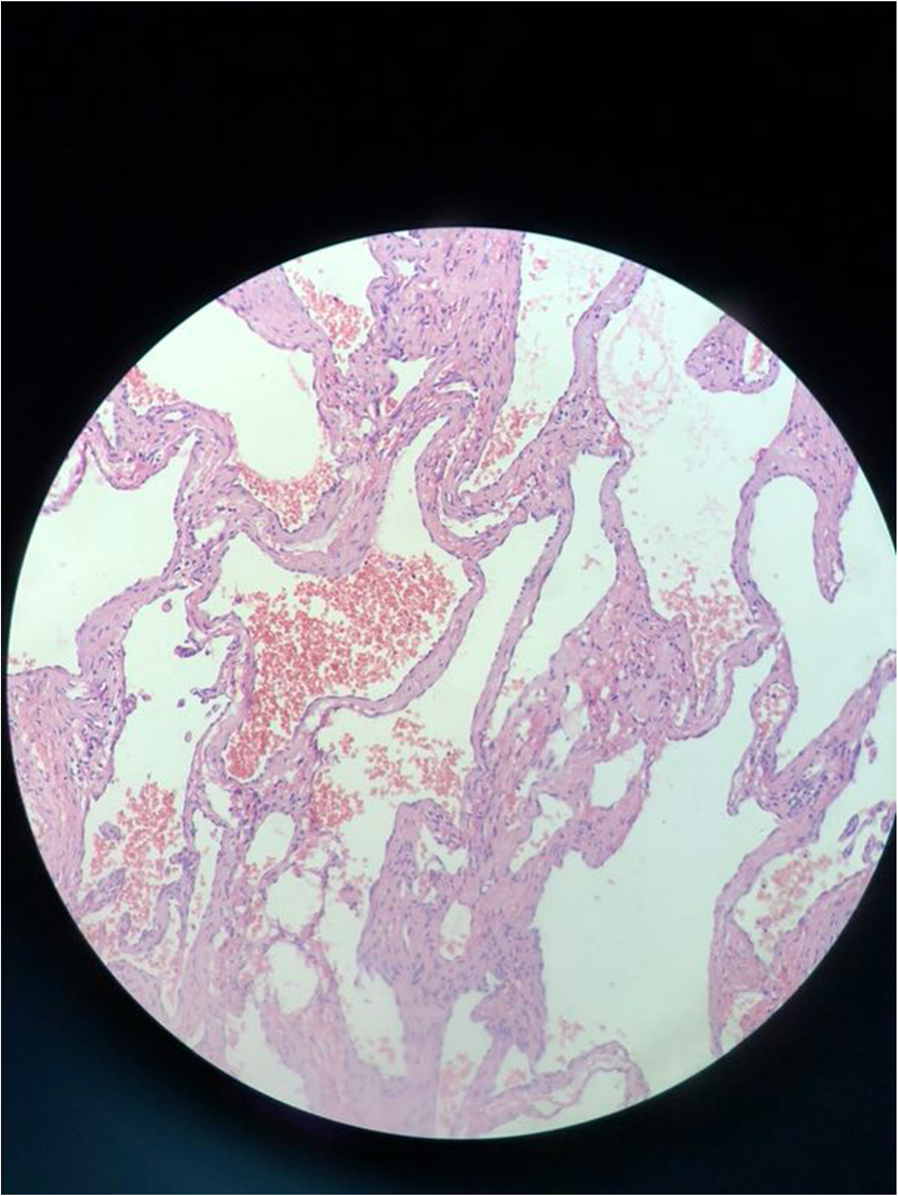
Histopathology examination (HPE) of the mass revealed multiple thin- and thick-walled dilated vessels, confirming the diagnosis of cavernous haemangioma

On day 5 of admission, patient underwent open surgery for tumour resection and mitral valve replacement. Median incision was performed, followed by cannulation of aorta, superior, and inferior vena cava. Cardiopulmonary bypass was established. Under moderate temperature and myocardial protection with cold blood cardioplegia, myocardial anterograde and retrograde cardioplegia perfusion through the aortic root and coronary sinus were established. A heart-shaped mass was visible on the surface of the anterior wall of the right ventricle. It was about 3 × 2 cm in size. The capsule was intact with a dark red appearance and tough texture. There was no adhesion to the surrounding tissue, and also no connection of blood vessels between the tumour and the heart, the haemangioma was nourished by general superfusion of blood. As a result, complete resection of the tumour was achieved with minimal difficulty. A patch of pericardium was sutured with 5–0 Prolene to repair the defect. Following that, the mitral valve was excised and the St Jude Regent No. 21 tissue valve was implanted. Post-operative histopathology examination (HPE) of the mass revealed multiple thin- and thick-walled dilated vessels, suggesting a cavernous type of haemangioma (Fig. 4).

Post-operatively, patient was observed in the hospital for 2 weeks for another neurological illness and subsequently discharged well on January 24th, 2018. The chest tightness and SOB improved significantly after the operation, resulting in better exercise tolerance. After 3 months, the patient returned to the hospital for follow up. There were no new complaints. No haemangioma recurrence was seen on CTA.

## Discussion

Cardiac haemangiomas share the same histological features with haemangiomas arising from other parts of the body. All haemangiomas are made up of benign proliferation of endothelial cells. Cardiac haemangiomas can originate from any layers of the heart or throughout the whole pericardium. Demographically, haemangiomas have been detected in almost all age group. However, there is a slight predominance among female patients. Some of the cardiac haemangiomas are asymptomatic and detected incidentally. However, depending on the anatomic site of the tumour and the extension of the tumour margin, patients with cardiac haemangioma may manifest different types of symptoms. Some patients may complain of educed exercise tolerance, dyspnoea, palpitation, atypical chest pain, and irregular heartbeats.

Among the different types of cardiac haemangiomas, cavernous haemangiomas are associated with slow growth rate and low invasion rate of the adjacent structures. Nevertheless, as a result of external compression, cavernous haemangiomas may manifest clinically as outflow tract obstruction, atrial compression, ventricular dysrhythmias, and embolization in the patient [4]**.**

Improvement in modern imaging modalities have greatly enhanced the diagnosis of cardiac haemangiomas. Traditionally, echocardiography is main investigation modality. Transoesophageal echocardiography is more useful than transthoracic echocardiography as it offers increased sensitivity in diagnosing cardiac haemangiomas [6]**.** However, echocardiography is invasive in nature and it also has certain limitations as it is not able to fully assess structures such as the mediastinum, diaphragm, and lung parenchyma that are distant to the transducer. A better modality would be CT as it is able to better demonstrate the tumour location, the relationship of the tumour with adjacent structures, and if there is any invasion into vital structures in the surrounding. Furthermore, CT may characterize the tumour lesion based on the attenuation values or pattern of enhancement in order to rule out other differential diagnoses. Another advantage of CT lies within its ability to determine if the tumour is confined regionally or it has metastasized to other distant structures.

In this patient, there was a rapid uptake of the iodine-based contrast by the highly vascular tumour during CT imaging. This proved to be useful in ruling out the intracardiac mass as a thrombus, as a thrombus would be non-enhancing in nature.

Nevertheless, magnetic resonance imaging (MRI) remains the gold standard of diagnosis of this condition as it provides a higher contrast resolution compared with CT. MRI is also able to detect any myocardial invasion apart from providing additional information about any functional effect the neoplasm might have incurred on the heart [7]**.** Last but not least, positron emission tomography (PET)/CT is another useful staging tool for cardiac haemangiomas as it can display any regional or distant spread of the tumours. However, its uptake is still low especially in financially constraint settings.

In this case, the patient was diagnosed as having a cardiac haemangioma. However, the site of origin of the tumour remained unclear. The natural history of cardiac cavernous haemangiomas is highly varied. With regard to the treatment modality, surgical excision is the preferred management for most surgeons. In view of the potential risk of embolism and rupture, it remains controversial to surgically remove all types of cardiac haemangiomas. However, it is almost universally accepted that symptomatic patients should undergo surgical resection. With adequate surgical resection, the long-term prognosis is often favourable. In contrast, patients who are undiagnosed and untreated may suffer from sudden death due to arrhythmias.

In short, cardiac haemangiomas are a type of rare but benign tumour which most commonly arise from the right side of the heart. These cardiac haemangiomas often have unpredictable natural histories and no specific clinical manifestations, resulting in diagnosis challenge. Despite so, imaging modality such as CT and MRI is a very useful in diagnosing and evaluating the surgical resectability of the haemangioma. Complete surgical excision remains the first line therapy. For majority of the patients, prognosis is generally favourable if detected and managed promptly.

## Data Availability

All data can be found at the first affiliated hospital of Dalian medical university, cardiothoracic surgery department.

## Abbreviations

CT scan: Computed tomography scan
CTA: Computed tomography angiography
HPE: Histopathology examination
MRI: Magnetic resonance imaging
PET: Positron emission tomography
